# Nitrogen Fixation Potential in Bathypelagic Sediment of the Ice‐Covered Arctic Ocean Revealed Through Long‐Term Stable Isotope Incubations

**DOI:** 10.1111/1758-2229.70173

**Published:** 2025-09-04

**Authors:** Lisa W. von Friesen, Carolin R. Löscher, Stefan Bertilsson, Hanna Farnelid, Pauline Snoeijs‐Leijonmalm, Marcus Sundbom, Sachia J. Traving, Flor Vermassen, Lasse Riemann

**Affiliations:** ^1^ Department of Biology, Marine Biology Section University of Copenhagen Helsingør Denmark; ^2^ Centre for Ecology and Evolution in Microbial Model Systems (EEMiS) Linnaeus University Kalmar Sweden; ^3^ Nordcee, Department of Biology University of Southern Denmark Odense Denmark; ^4^ Department of Aquatic Sciences and Assessment Swedish University of Agricultural Sciences Uppsala Sweden; ^5^ Department of Ecology, Environment and Plant Sciences Stockholm University Stockholm Sweden; ^6^ Department of Environmental Science (ACES) Stockholm University Stockholm Sweden; ^7^ Nordcee and HADAL, Department of Biology University of Southern Denmark Odense Denmark; ^8^ Department of Geological Sciences Stockholm University Stockholm Sweden

**Keywords:** benthic diazotrophs, deep‐sea, *nifH*, stable isotope‐tracing, sulphate‐reducing bacteria, sympagic‐pelagic‐benthic‐coupling

## Abstract

Due to climate change, sea ice more commonly retreats over the shelf breaks in the Arctic Ocean, impacting sea ice‐pelagic‐benthic coupling in the deeper basins. Nitrogen fixation (the reduction of dinitrogen gas to bioavailable ammonia by microorganisms called diazotrophs) is reported from Arctic shelf sediments but is unknown from the Arctic deep sea. We sampled five locations of deep‐sea (900–1500 m) surface sediments in the central ice‐covered Arctic Ocean to measure potential nitrogen fixation through long‐term (> 280 days) stable‐isotope (^15^N_2_) incubations and to study diazotroph community composition through amplicon sequencing of the functional marker gene *nifH*. We measured low but detectable nitrogen fixation rates at the Lomonosov Ridge (0.6 pmol N g^−1^ day^−1^) and the Morris Jessup Rise (0.4 pmol N g^−1^ day^−1^). Nitrogen fixation was observed in sediments with the lowest organic matter content and bacterial abundance, and where sulphate‐reducers like *Desulfuromonadia* and *Desulfosporosinus* sp. were prominent. Most *nifH* genes were distantly related to known diazotrophs. In this study, we show a potential for nitrogen fixation in Arctic bathypelagic sediments, considerably extending the known biome of marine nitrogen fixation. It raises the question of the significance of low but potentially widespread nitrogen fixation in deep‐sea sediments.

## Graphical abstract

We sampled five locations of deep‐sea surface sediments in the central ice‐covered Arctic Ocean and documented diazotroph communities and nitrogen fixation potential. The findings considerably extend the known biome of marine nitrogen fixation and raise the question of the significance of low but potentially widespread nitrogen fixation in deep‐sea sediments.

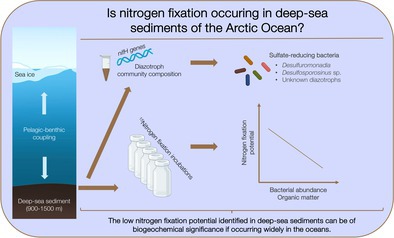

## 1 Introduction

Deep‐sea sediments harbour unique and highly diverse microbial communities (Jacob et al. 2013; Petro et al. 2017). Yet, our understanding of their activity and how they contribute to biogeochemical cycling is poor (Jørgensen and Boetius 2007). One important ecosystem function carried out by microbes is nitrogen fixation, which is performed by a group of microorganisms called diazotrophs. Diazotrophs reduce dinitrogen gas (N_2_) to ammonia (NH_3_), and their distribution and activity are important for understanding marine nitrogen and carbon cycling. Diazotrophs and nitrogen fixation have been increasingly acknowledged in deep‐sea habitats such as cold seeps (Chen et al. 2024), hydrothermal vents (Mehta et al. 2003), whale falls, and methane seeps (Dekas et al. 2018, 2009), but rarely in the “background sediments” (sediment far away from any such structures, instead relying on vertical flux from the water column) (Dekas et al. 2018; Hartwig and Stanley 1978) that cover most of the global open ocean deep seafloor. Nitrogen fixation in sediments is a poorly understood process in terms of both microbial ecology and biogeochemical impacts on local, regional, and global scales.

In the Arctic Ocean, nitrogen fixation has previously been estimated to 0.02–0.33 μmol N m^−2^ h^−1^ in shelf sediments of the Bering and Beaufort Seas by using the acetylene reduction method (Haines et al. 1981; Knowles and Wishart 1977). In shelf sediments of the Chukchi Sea, stable isotope tracer methodology (^15^N_2_) did, in contrast, not detect any nitrogen fixation (McTigue et al. 2016). In coastal northwest Svalbard (European Arctic), nitrogen fixation in Smeerenburgfjorden (0.83 μmol N m^−2^ h^−1^) was up to 20‐fold higher than in the Pacific Arctic studies (Bering and Beaufort Seas), while being undetectable further south in Svalbard (Gihring et al. 2010). More recently, diazotrophs were described from surface sediment in Kongsfjorden (Svalbard), being dominated by sulphate‐ and iron‐reducing *Deltaproteobacteria* (Jabir et al. 2021). So far, the mostly permanently sea ice‐covered deep basins (representing ~50% of the Arctic Ocean area) have, to the best of our knowledge, not been studied regarding benthic nitrogen fixation and/or diazotroph community composition.

The seasonal sea ice cover now regularly withdraws over the shelf‐break (Stevenson et al. 2023; Stroeve and Notz 2018), exposing underlying benthic ecosystems to increased deposition of organic material (Boetius et al. 2013; Bienhold et al. 2022). In other marine systems, sedimentation of organic matter influences benthic nitrogen cycling (Fulweiler et al. 2007; Capone 1988), but it is unknown how this factor specifically affects the Arctic deep‐sea benthic microbial communities. This hampers predictions of systemic responses to sea ice retreat and disappearance. Reported deep‐sea sediment nitrogen fixation elsewhere seems to be patchy, with higher rates in areas of elevated organic matter with low nitrogen content (such as methane seeps) (Dekas et al. 2018, 2014). The impact of complex organic matter (e.g., biomass from algae or metazoans) is less well known (Dekas et al. 2018) and deep‐sea nitrogen fixation has rarely been studied from a pelagic‐benthic coupling perspective. Increased deposition of organic matter to the Arctic deep seafloor due to sea ice decrease (Boetius et al. 2013) is predicted to cause elevated benthic remineralisation rates (Kiesel et al. 2020). By 2050, the central Arctic Ocean is predicted to become ice free in summer for the first time in thousands of years (Kim et al. 2023). Therefore, it is of high importance to constrain baseline benthic microbial processes to enable predictions and monitoring of the impact of sea ice disappearance on benthic microbial processes.

In this study, we investigated diazotroph community composition and measured the potential for nitrogen fixation in largely sea ice‐covered Arctic deep‐sea surface sediments. The study locations represented contrasting overlaying productivity regimes in rarely visited regions along the Lomonosov Ridge and the Morris Jessup Rise north of Kalaallit Nunaat (Greenland).

## 2 Experimental Procedures

### 2.1 Study Regions, Physicochemical Measurements, and Sampling

**FIGURE 1:**
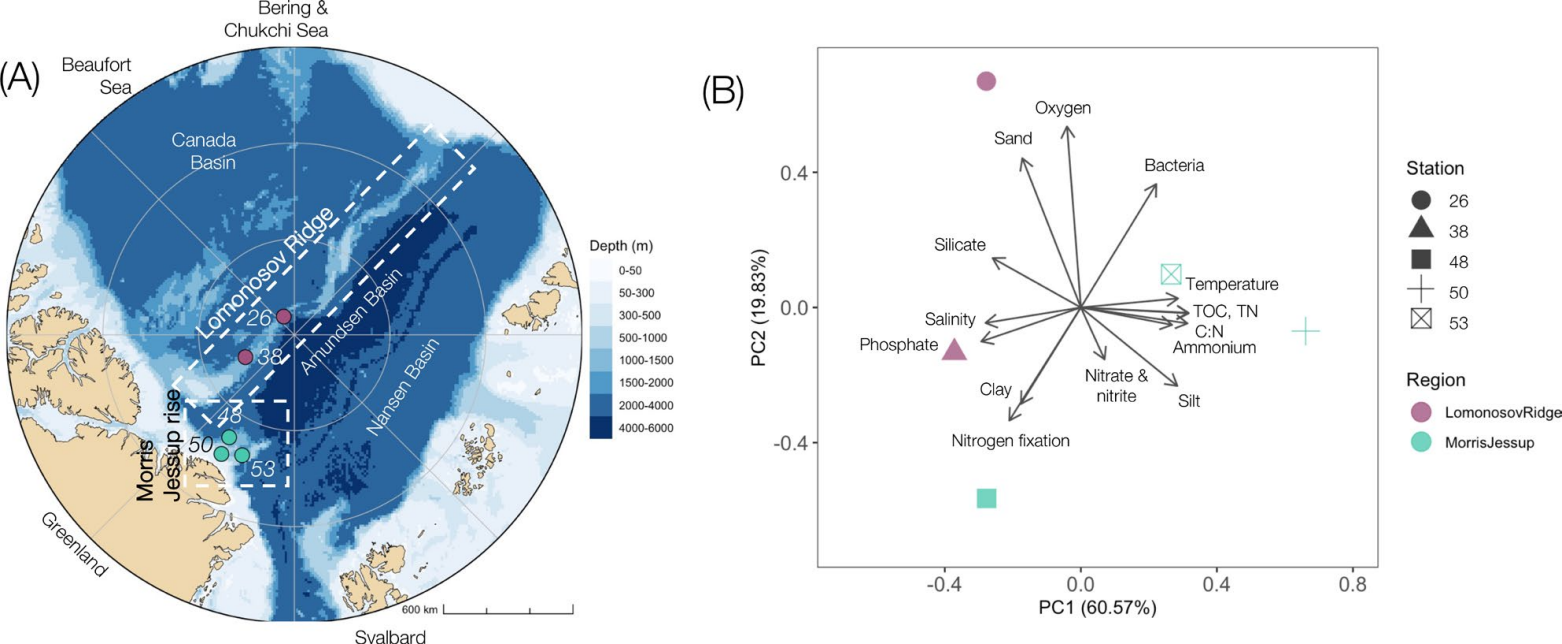
Map and environmental conditions of the study region. (A) Bathymetric map of the Arctic Ocean, depicting the positioning of sampling stations within the two regions Lomonosov Ridge and Morris Jessup Rise. Ridges, basins and regions of relevance to the study are labelled. Note that the map bathymetry around Station 50 is uncertain. (B) Principal component analysis of environmental conditions in sediment and water and measured nitrogen fixation rates of each station. The direction and length of arrows depict the loading of the variable. C:N: carbon to nitrogen molar ratio, TN: total nitrogen, TOC: total organic carbon.

Samples were collected onboard IB Oden from topographic highs in the central ice‐covered Arctic Ocean and the Wandel Sea during the Synoptic Arctic Survey expedition between 26 July and 19 September 2021. Two stations (Stations 26 and 38) were sampled along the Lomonosov Ridge between the Amundsen and Makarov basins (1187–1318 m), and three stations (Stations 48, 50 and 53) were sampled at the Morris Jessup Rise (901–1537 m depth) north of Greenland (Figure 1A; Table 1). Deep‐water currents at the Lomonosov Ridge originate from internal circulation in the adjacent basins, whereas the Morris Jessup Rise is influenced by an outflow of deep water from the Canadian, Makarov and Amundsen basins (Jones et al. 1995). The Morris Jessup Rise is known as part of “the last ice area” where thick, older sea ice was expected to survive the longest, but recent observations suggest rapid sea ice decline also in this region (Schweiger et al. 2021).

**TABLE 1:** Sampling stations and environmental conditions.

Station	26	38	48	50	53
Region	Lomonosov Ridge	Lomonosov Ridge	Morris Jessup Rise	Morris Jessup Rise	Morris Jessup Rise
Latitude	89°07.030′ N	87°45.084′ N	84°55.693′ N	84°10.092′ N	84°31.193′ N
Longitude	150°08.330′ W	66°24.329′ W	33°30.226′ W	32°28.905′ W	24°32.521′ W
Sampling date	2021‐08‐19	2021‐08‐29	2021‐09‐03	2021‐09‐05	2021‐09‐06
Sampling depth (m)	1318	1187	1537	901	1364
Harvesting date	2022‐06‐14	2022‐06‐14	2022‐06‐14	2022‐06‐14	2022‐06‐14
# Spiked incubations	6	3	4	4	5
# Control incubations	5	3	4	4	5
Sediment					
TOC (μmol g (dw)^−1^)	361	369	416	1706	1289
TN (μmol g (dw)^−1^)	63	66	71	268	210
C:N ratio (molar)	5.7	5.6	5.8	6.4	6.1
Bacterial abundance (10^7^ cells g (ww)^−1^)	4.39 ± 1.64	2.66 ± 1.42	2.91 ± 1.73	4.49 ± 5.05	4.42 ± 3.15
Water content (%)	60.0	52.4	61.8	62.4	59.6
Sand (%)	10.2 ± 3.2	7.6 ± 3.8	4.1 ± 2.3	4.53 ± 2.7	4.9 ± 5.4
Silt (%)	72.6 ± 2.0	73.6 ± 2.3	75.6 ± 0.8	79.6 ± 0.2	75.8 ± 4.6
Clay (%)	17.2 ± 2.4	18.7 ± 1.7	20.1 ± 3.4	15.9 ± 2.5	19.3 ± 1.0
δ^13^C (‰)	−21.20	−21.40	−21.20	−20.98	−21.38
δ15N (‰)	0.37066	0.37057	0.37058	0.37062	0.37057
Device operation	SO21_26‐06	SO21_38‐12	SO21_48‐06	SO21_50‐15	SO21_53‐11
Seawater (from CTD, ~5–10 m above bottom)					
Oxygen (μmol kg^−1^)	298.8	294.7	293.8	294.6	296.5
Temperature (°C)	−0.3	−0.2	−0.3	−0.1	−0.1
Salinity	34.914	34.907	34.917	34.890	34.889
Silicate (μM)	8.46	8.60	8.12	7.28	8.36
Nitrate+Nitrite (μM)	12.97	12.55	13.42	12.99	13.28
Phosphate (μM)	0.91	0.89	0.96	0.75	0.77
Ammonium (μM)	0.00	0.00	0.01	0.56	0.02

Abbreviations: Bacteria: bacterial abundance, C:N ratio: carbon to nitrogen molar ratio, TN: total nitrogen, TOC: total organic carbon.

Sediment was collected with a box corer (0.5 × 0.5 m; one core per station). The sediment grain size was measured with a Malvern Mastersizer 3000 (Malvern Panalytical, UK), and the data were analysed using the software Gradistat (Blott and Pye 2001). The water content of the sediments (top 1 cm) was determined by weighing the sample before and after freeze‐drying.

Temperature, salinity and oxygen concentration of the water column were recorded by calibrated CTD sensors (Sea‐Bird, SBE 911+) (Table 1) (Heuzé et al. 2022) and seawater was collected with Niskin bottles at the maximum sampling depth of each station (5–10 m above the bottom) for analysis of inorganic nutrients. Unfiltered seawater, sampled from the Niskin bottles using clean methods, was photometrically determined onboard with a continuous segmented flow analyser (Seal QuAAtro39) within a day of sampling.

### 2.2 Flow Cytometry for Cell Enumeration

Samples from the sediment cores were collected (*n* = 2 per horizon) from the surface and ~2 cm below the surface in 2 mL cryotubes with a sterile syringe, fixed with glutaraldehyde (final concentration 1%; Sigma Aldrich, MA, USA), and incubated at room temperature for 20 min until frozen at −80°C. Samples were defrosted and weighed before being processed as described previously (Schauberger et al. 2021) with the following modifications: volumes were adjusted to the volume of sediment (~2 mL), all steps used artificial seawater (in 1 L MilliQ water: 2.35 g NaCl, 1.07 g MgCl_2_•6H_2_O, 0.907 g Na_2_SO_4_•10H_2_O, 0.146 g CaCl_2_•2H_2_O, 0.066 g KCl, 0.02 g NaHCO_3_, 0.003 g H_3_BO_3_), and three rounds of washing were done with 5 mL each time. Samples were stained using SYBR Green I (Sigma Aldrich, Germany) and measured on a BD FACSCanto II (BD BioSciences, USA).

### 2.3 Long‐Term Incubations for Nitrogen Fixation Rate Measurements

The incubation setup is outlined in Figure S1. Seawater overlying the sediment core was siphoned off. Two litres of the seawater were sterile‐filtered (0.22 μm, Sterivex, Millipore, MA, USA), and half of this volume was spiked with ^15^N_2_ (5.5 mL L^−1^; 99%, Cambridge Isotope Labs, MA, USA; LOT number 1‐24583/AR0664758), and repeatedly shaken for 30 min at 4°C. From the isotope‐spiked water, 12 mL (*n* = 2) was collected in exetainers (Labco, Lampeter, UK) for quantification of the ^15^N atom% enrichment of the dissolved N_2_ pool (average 32.3%) with membrane inlet mass spectrometry (Kana et al. 1994) (Data S1). Surface sediment (0–4 cm, previously shown to have higher nitrogen fixation activity (Dekas et al. 2018)) was collected with a sterile plastic spoon into an acid‐washed glass beaker, and 1 mL subsamples of sediment were collected and frozen at −20°C for the analysis of total organic carbon (TOC) and total nitrogen (TN) content (*n* = 1) and DNA (*n* = 1) (see below for both methods). The remaining sediment was gently mixed 1:1 (v/v) with the filtered seawater (half of the replicates with isotope‐spiked water, half of the replicates without; Figure S1) under a stream of N_2_ (100624688, Linde, Solna, Sweden) to minimise O_2_ exposure (except for Station 26 where N_2_ was not available). Acid‐washed glass serum vials (60 mL) were flushed with N_2_ for 30 s, gently filled with the homogenised sediment slurry, and the headspace flushed with N_2_ before being crimp sealed with butyl rubber stoppers and aluminium caps and incubated in darkness while fully submerged in water at 4°C (i.e., about 4°C higher than overlaying seawater in situ) for 281–299 days (Table 1). Based on a previous study on deep‐sea nitrogen fixation, several months of incubation is recommended due to the low microbial activity (Dekas et al. 2009). We acknowledge that long incubation times may introduce biases in tracer signals but account for this by using parallel control incubations (identical incubations except for the addition of ^15^N_2_). Upon termination of the incubations, water was gently siphoned off, the sediment was homogenised with a sterilised spatula, and samples were collected with a sterile syringe for the analysis of isotopic enrichment with elemental‐analyser to isotope ratio mass spectrometry (EA‐IRMS) and DNA. The samples were frozen at −20°C. Isotopic enrichment (^15^N/^14^N) was determined after acid fuming (using concentrated HCl in a desiccator overnight, followed by a drying step of 12 h at 50°C) using an elemental analyser (Flash EA 1112 series, Thermo Fisher, USA) coupled to an isotope ratio mass spectrometer (Finnigan Delta Plus XP, Thermo Fisher, USA) against a caffeine standard (Reeder et al. 2022). Nitrogen fixation rates (pmol N g (dw)^−1^ day^−1^) (Montoya et al. 1996) and error propagation‐based limit of detections (LOD) (Gradoville et al. 2017) were calculated using the template provided by White et al. (2020), modified for the calculation of rates per gram dry weight sediment (Data S1). Natural ^15^N/^14^N isotope ratios were averages of the control incubation vials from each station (*n* = 3–5). It is challenging to simulate in situ pressure and temperature conditions of the deep‐sea environment. We acknowledge this as a methodological drawback and, therefore, discuss the measured nitrogen fixation rates as potential rates. Future technical advancement should aim to enable in situ incubations for representative deep‐sea measurements, avoiding alteration of sediment microstructures and physicochemical parameters through resuspension and mixing, and bottle effects during long‐term laboratory incubations.

### 2.4 Amplicon Sequencing of the Nitrogenase Gene (*nifH*)

DNA was extracted from 0.5 mL of thawed sediment with the DNeasy PowerSoil Pro kit (Qiagen, Hildesheim, DE) with four additional steps as described in (Kapili et al. 2020) before bead beating for 5 min: centrifugation at 10.000 g for 30 s, removal of the supernatant, vortexing with CD1 solution, and heating for 10 min at 65°C (briefly vortex after 5 min). The *nifH* gene amplicons were generated in a nested PCR reaction (Zehr and Turner 2001) applying primers nifH1‐nifH4 (Table S1), and sequencing libraries were prepared as previously described (von Friesen et al. 2023). Duplicate negative controls (PCR grade UV‐irradiated water as template) were included for sequencing. Sequencing was performed on an Illumina MiSeqV3 platform (2 × 300 bp; Geogenetics, University of Copenhagen, Denmark). Generation, quality control and taxonomic assignment of amplicon sequence variants (ASVs) were performed as in von Friesen et al. (2023). Briefly, we used (1) DADA2 (v.1.32.0) (Callahan et al. 2016) for inference of ASVs (trimming: 240 forward, 180 reverse), (2) parts of the NifMAP pipeline to exclude potential *nifH* homologues (v.1.0) (Angel et al. 2018), (3) assignment of *nifH* phylogenetic clusters with classification and regression trees (Frank et al. 2016), and (4) taxonomic assignment with the assignTaxonomy function of DADA2 applying a *nifH* database (Moynihan and Reeder 2023; Heller et al. 2014). After these steps, 935 ASVs were obtained from the 32 environmental and 2 negative control samples, with 55,368 and 33 average reads per sample, respectively. ASV sequences, taxonomic table including *nifH* clusters, and read abundance tables are available in Data S2A–E. To remove potentially contaminating ASVs, the R‐package decontam (v.1.24.0) (Davis et al. 2018) was applied with the prevalence method (default settings), resulting in the removal of one ASV (ASV51). The *nifH* gene has been questioned as an explicit marker gene for nitrogen fixation (Mise et al. 2021). Uncertainties concerning pseudo‐*nifH* sequences are mainly for species in the class *Clostridia*, which in this study made up 10% of the total relative abundance, and this taxon should thus be interpreted with caution in terms of their nitrogen fixation potential. A maximum‐likelihood phylogenetic tree was constructed on translated *nifH* sequences in raxmlGUI (v.2.0.10, (Edler et al. 2021), L‐INS‐I alignment, LG, G4 gamma mean, 100 bootstraps) and visualised with iTOL (https://itol.embl.de; Letunic and Bork 2021).

### 2.5 Data Analysis

Statistical analyses were performed with R (v.4.4.0), and data visualised with ggplot2 (v.3.5.1) (Wickham 2016) and ggOceanMaps (v.1.3.4) (Vihtakari 2022). Initial pre‐processing of *nifH* ASVs was performed in phyloseq (v.1.48.0) (McMurdie and Holmes 2013), where nine singletons were removed. Rarefaction curves were generated to confirm sufficient sequencing depth. No rarefaction was performed. ASV count tables were centred log ratio‐transformed (microbiome; v.1.26.0) (Lahti and Sudarshan 2017) for principal component analyses (PCA; covariance matrix) and redundancy analysis (RDA) using Aitchison distance. Permutational multivariate analysis of variance (999 permutations) with adonis2 on Aitchison distance matrices was used to evaluate categorical differences. To ensure homogeneity of group dispersions, the function permutest of the function betadisper was used in parallel to adonis2. Eventual correlations between environmental variables and nitrogen fixation rates were explored through PCA and Spearman's rank correlation on *z*‐scored data. The code used for the study is provided in Data S3.

## 3 Results

### 3.1 Regional Biogeochemical Characteristics

The sampling stations were located in contrasting regions with thick overlaying multiyear ice (up to 3.2 m) at Stations 26, 38 and 48 and partly open water at Stations 50 and 53 on the Morris Jessup Rise (open drift of single‐ and multiyear ice) (Snoeijs‐Leijonmalm 2022). Note that dense sea ice has covered the Morris Jessup Rise until recently (Schweiger et al. 2021). TOC and TN were low in sediments at Stations 26, 38 and 48 but up to three to five times higher at Stations 50 and 53 (Figure 1B; Table 1). The carbon‐to‐nitrogen molar ratios ranged between 5.6 and 6.4 (i.e., below the Redfield ratio) (Table 1), and ∂^13^C was above −25‰, which are values characteristic of marine origin (Kim et al. 2020). The sediments were predominantly composed of silty clay with a high water content (Table 1). Bacterial abundances were about two times lower at Stations 38 and 48 compared to the other stations (Figure 1B; Table 1).

Deep seawater above the sediments was of similar temperature (average −0.2°C ± 0.12°C), salinity (average 34.904 ± 0.013) and well‐oxygenated (average 295.7 ± 2.0 μmol O_2_ kg^−1^) across stations (Table 1). Inorganic nutrients were well above detection limit in deep seawater at all stations, but ammonium was only quantifiable at the three Morris Jessup Rise stations (Figure 1B; Table 1).

Station 48 (northern Morris Jessup Rise) was, based on principal component analysis of all environmental variables, more similar to the Lomonosov Ridge stations (26 and 38) than to the other Morris Jessup Rise stations (50 and 53). This was driven by its marginally higher salinity and phosphate concentration, and lower TOC and TN concentrations than Stations 50 and 53 (Figure 1B; Table 1).

### 3.2 Magnitude of Nitrogen Fixation

**FIGURE 2:**
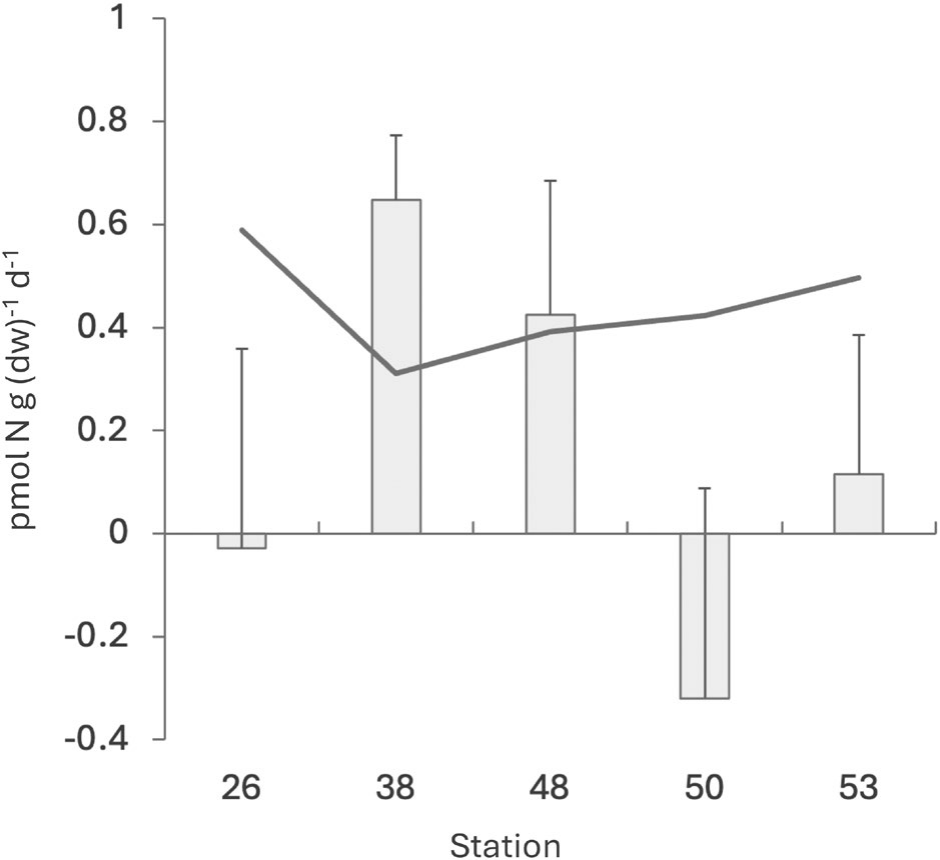
Potential nitrogen fixation rates. Average nitrogen fixation rates (pmol N g (dw)^−1^ day^−1^) measured for the different stations. The solid black line depicts the limit of detection for each station. Error bars denote one standard deviation. *n* = 6, 3, 4, 4, 5 at Stations 26, 38, 48, 50, 53, respectively. Negative values may indicate other microbial processes than nitrogen fixation altering the isotopic fractionation.

Nitrogen fixation was detected in sediment from two of the five stations: Stations 38 (western Lomonosov Ridge; 0.65 ± 0.13 pmol N g (dw)^−1^ day^−1^; above the level of detection in 3/3 replicates) and 48 (northern Morris Jessup Rise; 0.42 ± 0.26 pmol N g (dw)^−1^ day^−1^; above the level of detection in 2 out of 4 replicates) (Figure 2). The level of detection ranged from 0.31 to 0.72 pmol N g (dw)^−1^ day^−1^ (Figure 2). Nitrogen fixation was associated with stations of clay‐rich sediment, higher phosphate concentrations and salinity, and lower C:N molar ratio, ammonium, and TOC (Figure 1B). Through Spearman correlation analysis, nitrogen fixation was identified to be negatively correlated with bacterial abundance (spearman, rho = −0.89, *p* = 0.04). No other statistically significant correlations were observed.

### 3.3 Diazotroph Community Composition

**FIGURE 3:**
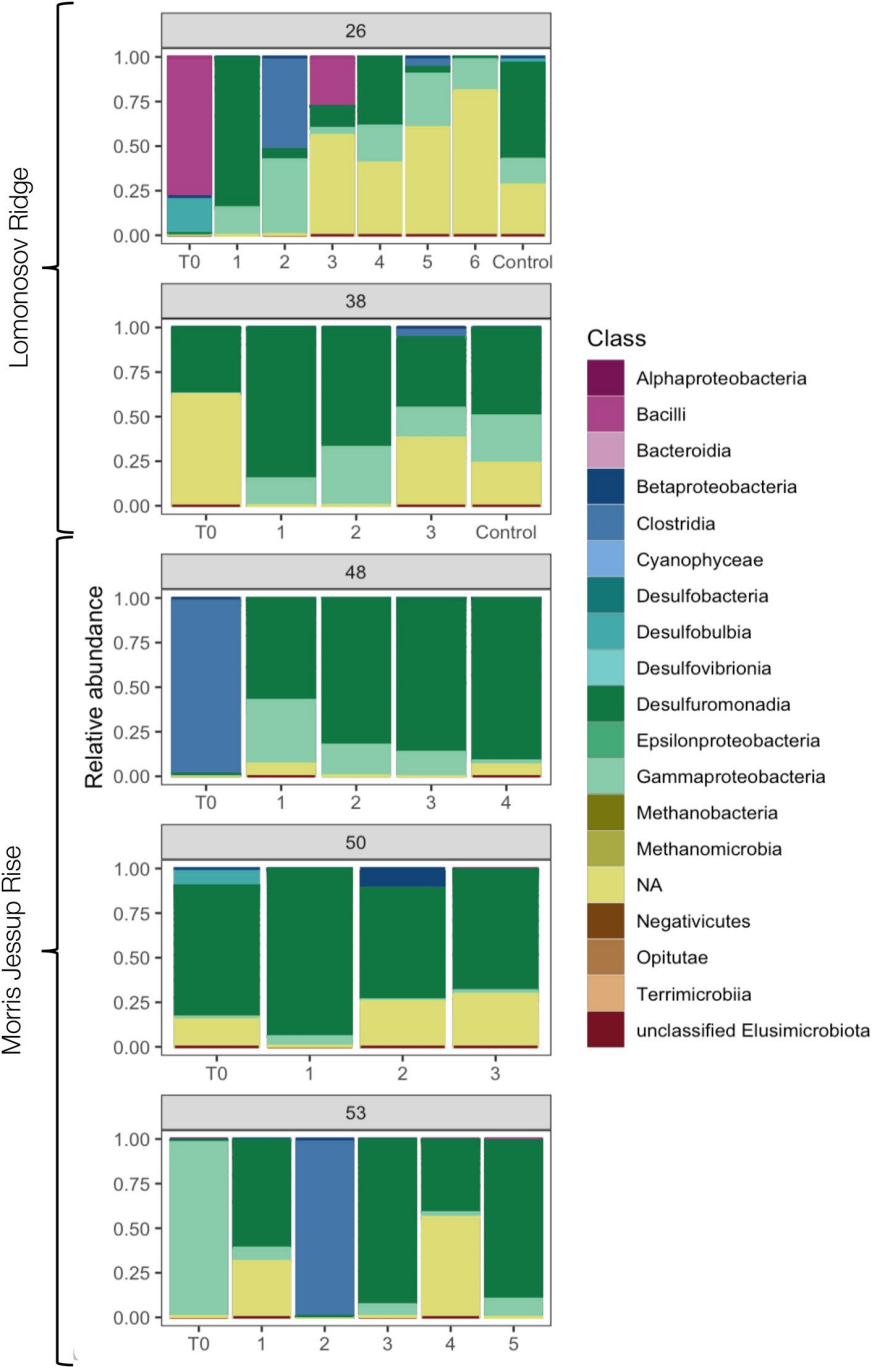
Diazotroph community composition. The relative abundance of diazotroph classes at each station was assessed through *nifH* amplicon sequencing (3–6 replicates per station after the long‐term incubation, ranging from 281 to 299 days). “T_0_” at each station shows the diazotroph community composition at the time of sampling for the start of the incubation. Samples from two control incubations (incubation without ^15^N_2_) were also sequenced (from Stations 26 and 38). NA: no assigned taxonomy at class level.

The composition of benthic diazotrophs differed markedly between the stations (*T*_0_), with the highest relative abundances of class *Bacilli* (*Anaerobacillus* sp.; 78.2%) at Station 26, an unknown class (Subcluster 3N; 62.3%) and *Desulfuromonadia* (37.1%) at Station 38, *Clostridia* (*Desulfosporosinus* sp.; 98.8%) at Station 48, *Desulfuromonadia* (73.9%) at Station 50, and *Gammaproteobacteria* (*Ketobacter* sp.) (97.8%) at Station 53 (Figure 3). ASVs in the *T*_0_ sediment samples were from phylogenetic *nifH*‐clusters 1 (77.1%), 3 (22.9%), and 2 (0.006%). No archaeal *nifH* sequences were detected in the *T*_0_ sediment samples, and they represented only 0.01% of total reads (*Methanobacteria* and *Methanomicrobia*). The diazotroph community composition in overlying seawater samples (Station 38 not included due to missing sample) indicated a difference compared to the sediments (adonis2, *R* ^2^ = 0.18, *p* = 0.085) where several classes (e.g., *Bacteroidia* and *Desulfovibrionia*) were relatively more abundant in the water than in the sediment (Stations 48 and 50) (Figure S2). Diazotrophs associated with nitrogen fixation (based on nitrogen fixation in individual vials and their corresponding DNA sample) were *Gammaproteobacteria* (order *Alteromonadales*; Subcluster 1G) and *Thermodesulfobacteriota* (Subcluster 3E) (Figure S3).

Of the 20 ASVs with the highest relative abundance (collectively representing 55.7% of all reads), ASV1, ASV3, and ASV10 could not be assigned taxonomy (Data S2). Blastn analysis (National Center for Biotechnology Information NCBI) revealed similarities to previously reported *nifH* genes from deep‐sea sediments of the South China Sea (1548 m, 91.9% (ASV 1), 95.4% (ASV3), HQ223710, Dang et al. 2013) and from seawater of the sediment‐influenced Northern Benguela upwelling system (100 m, 89.1% (ASV10), OK083133, Reeder et al. 2022) (Figure S4). ASV1 was also found to be 85.9% similar to a *nifH* gene fragment reported from sediment and/or algae‐laden Arctic sea ice (“brown ice”) in the Amundsen basin of the central Arctic Ocean (KT354146, Fernández‐Méndez et al. 2016) (Figure S4). ASV1 was in our study recovered at all stations except Station 50 (most abundant at Stations 26 and 53). Aligning all top 20 ASVs against a recently compiled nucleotide database of non‐cyanobacterial diazotrophs (Turk‐Kubo et al. 2023) identified ASV19 (*Desulfuromonadales*) as 96.3% similar to an operational taxonomic unit (OTU) from the Chukchi Sea (OTU4, Pacific Arctic), where it was speculated to be of sediment origin (Shiozaki et al. 2018). In our study, ASV19 was found in sediments from Stations 26 (1.4% after incubation), 50 (up to 31.1% after incubation), and 53 (0.19% at *T*_0_, up to 15% after incubation) but not from Stations 38 and 48. Further, the search revealed the presence of “Gamma 4” (Halm et al. 2012) (ASV14, 100% nucleotide similarity) in the sediment (*T*_0_) from Stations 38 and 53 (0.3% and 68.8%, respectively) and in seawater from Stations 26, 50 and 53 (0.01%, 0.06% and 0.82%, respectively).

## 4 Discussion

The data presented here considerably extends the potential habitat for marine nitrogen fixation to also include bathypelagic Arctic sediments and raises the question of the ecological and biogeochemical importance of low but potentially widespread nitrogen fixation rates in deep‐sea sediments.

### 4.1 Potential Nitrogen Fixation Rates in Deep‐Sea Arctic Sediment

Our data show that there is a potential for nitrogen fixation in Arctic bathypelagic sediments. The potential nitrogen fixation rates suggested by our long‐term laboratory incubations were associated primarily with environments with low pelagic‐benthic coupling (i.e., thick perennial sea‐ice cover and low organic matter content in the sediment). The nitrogen fixation rates are low relative to previous findings, being one order of magnitude lower than in North Atlantic sediments (5.16 pmol N g (dry weight)^−1^ day^−1^) (Hartwig and Stanley 1978) and three orders of magnitude lower than in North Pacific sediments (218.4 pmol N g (dry weight)^−1^ day^−1^) (Dekas et al. 2018). However, the comparability to previous studies is complicated by different methodologies, target depths (bottom depth and sediment depth below surface), and environmental settings (e.g., organic matter content). While our reported rates are low, the potential occurrence of nitrogen fixation over larger seafloor areas may still significantly influence benthic microbial ecology, biogeochemistry, and solute exchange.

Nitrogen fixation in Arctic bathypelagic surface sediments was associated with locations with low TOC and TN concentrations and low C:N molar ratios (relative to the stations where nitrogen fixation was not detected). Due to our limited dataset and the potential for multiple environmental factors regulating benthic nitrogen fixation, we hesitate to conclude a potential suppression of benthic nitrogen fixation by organic matter in the Arctic Ocean. Rather, our findings highlight a need for deciphering the importance of quality and quantity of organic matter for benthic nitrogen fixation—a need evident for coastal and deep‐sea sediments alike (Dekas et al. 2018; Fulweiler et al. 2007). The recent break‐up and export of sea ice in the Wandel Sea (Schweiger et al. 2021) may be one reason for the relatively higher concentrations of TOC and TN (von Jackowski et al. 2023) measured in sediments of Stations 50 and 53, possibly indicating deposition of fresh pelagic (and/or sympagic; Boetius et al. 2013) organic matter. It should also be noted that Station 50 was the shallowest station (901 m). A re‐occurring relationship between Arctic phytoplankton blooms and elevated sediment bacterial abundance was recently described (Ramondenc et al. 2024). Here, in support of phytodetritus being the source of the organic matter at Stations 50 and 53, sediment bacterial abundance was two times higher compared to the still permanently sea‐ice‐covered Station 48 at the northern Morris Jessup Rise. Bacterial abundance was also negatively correlated with observed nitrogen fixation. It is unknown when a potential deposition might have taken place and at what stage the eventual decomposition process was at the time of sampling. However, it has been suggested that heterotrophic diazotrophs gain a competitive advantage once nitrogen is depleted while organic carbon is still available, a situation expected to occur late in the decomposition process of organic matter (Riemann et al. 2022; Chakraborty et al. 2021). At Stations 38 and 48, where nitrogen fixation was detected, the TOC, TN and C:N ratios were lower (< 6). As nitrogen fixation is an energetically costly process that is usually constrained in nitrogen‐replete environments (Knapp 2012), its occurrence at low C:N ratios may indicate that the present nitrogen is not readily available to the diazotrophs. Overall, nitrogen fixation in central Arctic Ocean sediment was in our study associated with locations with lower organic matter content and lower bacterial abundances.

### 4.2 Deep‐Sea Arctic Diazotrophs

We find that phylogenetically and physiologically diverse diazotrophs with low similarity to previously reported diazotrophs reside in Arctic bathypelagic sediments, suggesting a potential for nitrogen fixation under different biogeochemical conditions. In methane‐rich deep‐sea seep environments, diazotrophic sulphate‐reducing bacteria thrive in consortia with anaerobic methanotrophic archaea (Dekas et al. 2009), whereas in the North Pacific, free‐living sulphate‐reducing heterotrophic bacteria are suggested candidate diazotrophs in background sediments (i.e., no known specific organic carbon source except pelagic export) (Dekas et al. 2018). In line with the Pacific background sediments, we retrieved several anaerobic sulphate‐reducing groups (e.g., *Desulfuromonas*, *Desulfobulbia*, *Desulfosporosinus* sp., and *Ketobacter* sp.) and no archaeal *nifH* gene sequences, supporting the potential importance of sulphate‐reducing diazotrophs in low‐carbon sediments. To further establish the diazotrophs that are responsible for the nitrogen fixation in deep‐sea sediments, e.g., expression analyses are needed.

At the stations where nitrogen fixation was detected, *Clostridia* (Station 48, *Desulfosporosinus* sp.), *Desulfuromonadia*, and an unknown Cluster 3N diazotroph (Station 38; ASV10) were the dominant groups. The metabolism of ASV10 remains unknown, but Cluster 3 diazotrophs include anaerobic sulphate‐reducers (Zehr et al. 2003). It is unknown which diazotrophs were actively fixing nitrogen, and future studies targeting the expression and/or activity of specific diazotrophs are warranted (Li et al. 2024). Overall, sulphate‐reducing bacteria (*Desulfuromonadia*, *Desulfosporosinus* sp., and Cluster 3N) are candidate key diazotrophs in Arctic bathypelagic sediments, and their potential activity in situ should be confirmed. We encourage metagenome‐assembled genomes and metatranscriptomes to guide future studies and leverage challenges (e.g., uncertainties with inferring nitrogen fixation potential and activity, taxonomy and potential horizontal gene‐transfer processes) associated with *nifH*‐based studies.

We report Gamma 4 (*Ketobacter* sp.) in deep‐sea sediments from the Wandel Sea, largely expanding its known biogeography. A previously metagenome‐assembled genome of Gamma 4 from bathypelagic seawater implies that they are particle‐associated and capable of not only nitrogen fixation but also nitrate reduction and denitrification with a complex role in the nitrogen cycle (Acinas et al. 2021). It is unknown whether they are advected from the Pacific or if there is a sustained but so far undescribed Arctic population. Gamma 4 is suspected to be associated with eukaryotic cells (mainly dinoflagellates), either as a facultative symbiont or as prey (Cheung et al. 2021), and may be exported with such hosts to the seafloor. In general, sinking marine snow can export diazotrophs (Turk‐Kubo et al. 2023) and Arctic algae can export bacteria to the deep seafloor (Rapp et al. 2018). Interestingly, our unknown ASV1 (Subcluster 1A) had low similarity to all records of the “core_nt” database of NCBI [2024‐10‐15], with one of the highest similarities (85.9%) being to an OTU from sediment and/or algae‐laden sea ice in the central Arctic (Fernández‐Méndez et al. 2016). Further, we recovered some cyanobacterial *nifH* sequences in the sediments of Stations 50 and 53 (*Halotia* sp. 1.2% relative abundance, and *Xenococcus* sp. 0.15%, respectively). Taken together, this indicates that sea ice may not only be a transport vector of microorganisms across the Arctic Ocean (von Friesen et al. 2023; Fernández‐Méndez et al. 2016) but also, upon melting, constitutes a potential source for downward transport of released sympagic organic matter. Overall, it is unknown which diazotrophs are specific to the sediment, to sinking particulate matter, or are simply exported from surface communities, but the sympagic‐pelagic‐benthic coupling seems important for a wider understanding of diazotrophs and nitrogen fixation in the Arctic Ocean.

We specifically targeted a multiyear ice‐covered region as this biome is rapidly disappearing (Kim et al. 2023), with an altered pelagic‐benthic coupling expected to impact microbial communities and benthic nitrogen cycling. Our study shows a potential for nitrogen fixation in bathypelagic ice‐covered Arctic sediments—a biome for which nitrogen fixation has not been previously reported. Our findings further support the need for studying nitrogen fixation in deep‐sea environments for understanding the global nitrogen cycle. Our study also provides a diazotroph biodiversity reference for future studies in the wake of climate change, reporting previously unknown diazotrophs. We encourage future studies of benthic microbial processes, including nitrogen fixation, to perform replicated and multiple time‐point sampling beyond the topographic highs of the bathypelagic Arctic. We further recommend experimentally manipulated incubations with added organic matter to central Arctic sediments (see, e.g., Li et al. 2024; Albert et al. 2021; Sen et al. 2024), simulating an open water situation that will soon be the new Arctic.

## Author Contributions

L.W.F., H.F., and L.R. designed the study. L.W.F. and H.F. collected the samples. C.R.L. performed EA‐IRMS analyses. L.W.F. performed the molecular analyses. F.V. performed grain size and water content analyses. S.J.T. designed and performed flow cytometry analyses. M.S. performed inorganic nutrient analyses. L.W.F. executed the data analysis and drafted the manuscript with L.R. and C.R.L. All authors edited the manuscript and approved the final version.

## Conflicts of Interest

The authors declare no conflicts of interest.

## Supporting information


**Data S1:** Nitrogen fixation rate calculations. Datasheet of nitrogen fixation rate calculations and the determination of error propagation‐based limits of detection.

**Data S2:** ASV‐related information. (A) Generated *nifH* amplicon sequence variants (nucleotides), (B) generated *nifH* amplicon sequence variants (amino acids), (C) taxonomic table, (D) read abundance table, (E) key for sample identifiers.

**Data S3:** Code. R code used for the data analysis and visualisation.

**Data S4:** Supporting Information.

## Data Availability

The data that supports the findings of this study are available in the Supporting Information of this article. The raw fastq files for *nifH* sequences of this study are openly available in the Sequence Read Archive of NCBI (National Center for Biotechnology Information) at [https://www.ncbi.nlm.nih.gov/bioproject/PRJNA1154448], reference number PRJNA1154448. The code used for the analyses and the generated amplicon sequence variants are available in the Supporting Information of this article.
