# Supplementary material for: Nitrogen Fixation Potential in Bathypelagic Sediment of the Ice‐Covered Arctic Ocean Revealed Through Long‐Term Stable Isotope Incubations
Source: Environ Microbiol Rep. 2025 Sep 4;17(5):e70173. doi: 10.1111/1758-2229.70173 (PMC12410090; doi:10.1111/1758-2229.70173)
Supplement: Supplementary file 4 — Data S4: Supporting Information. [file EMI4-17-e70173-s003.docx]

Supplementary figures and tables

Supplementary figures S1-S4 and supplementary table S1 to the article:

**“Nitrogen fixation potential in bathypelagic sediment of the ice-covered Arctic Ocean revealed through long-term stable isotope incubations”**

von Friesen L.W.*, Löscher, C. R., Bertilsson, S., Farnelid, H., Snoeijs-Leijonmalm, P., Sundbom, M., Traving, S.J., Vermassen, F. & Riemann, L.

*corresponding author: [lisa.winbergvonfriesen@lnu.se](mailto:lisa.winbergvonfriesen@lnu.se)

In addition, the following three data files are available as supplementary files:

Data S1. **Nitrogen fixation rate calculations.** Datasheet of nitrogen fixation rate calculations and the determination of error propagation-based limits of detection.

Data S2. **ASV-related information.** (A) Generated *nifH* amplicon sequence variants (nucleotides), (B) generated *nifH* amplicon sequence variants (amino acids), (C) taxonomic table, (D) read abundance table, (E) key for sample identifiers.

Data S3. **Code.** R code used for the data analysis and visualisation.

Figure S1. **Schematic drawing of the incubation setup.** FCM: flow cytometry for bacterial abundance, EA-IRMS: elemental analyser isotope ratio mass spectrometry, MIMS: membrane-inlet mass spectrometry. Produced with icons from Biorender.


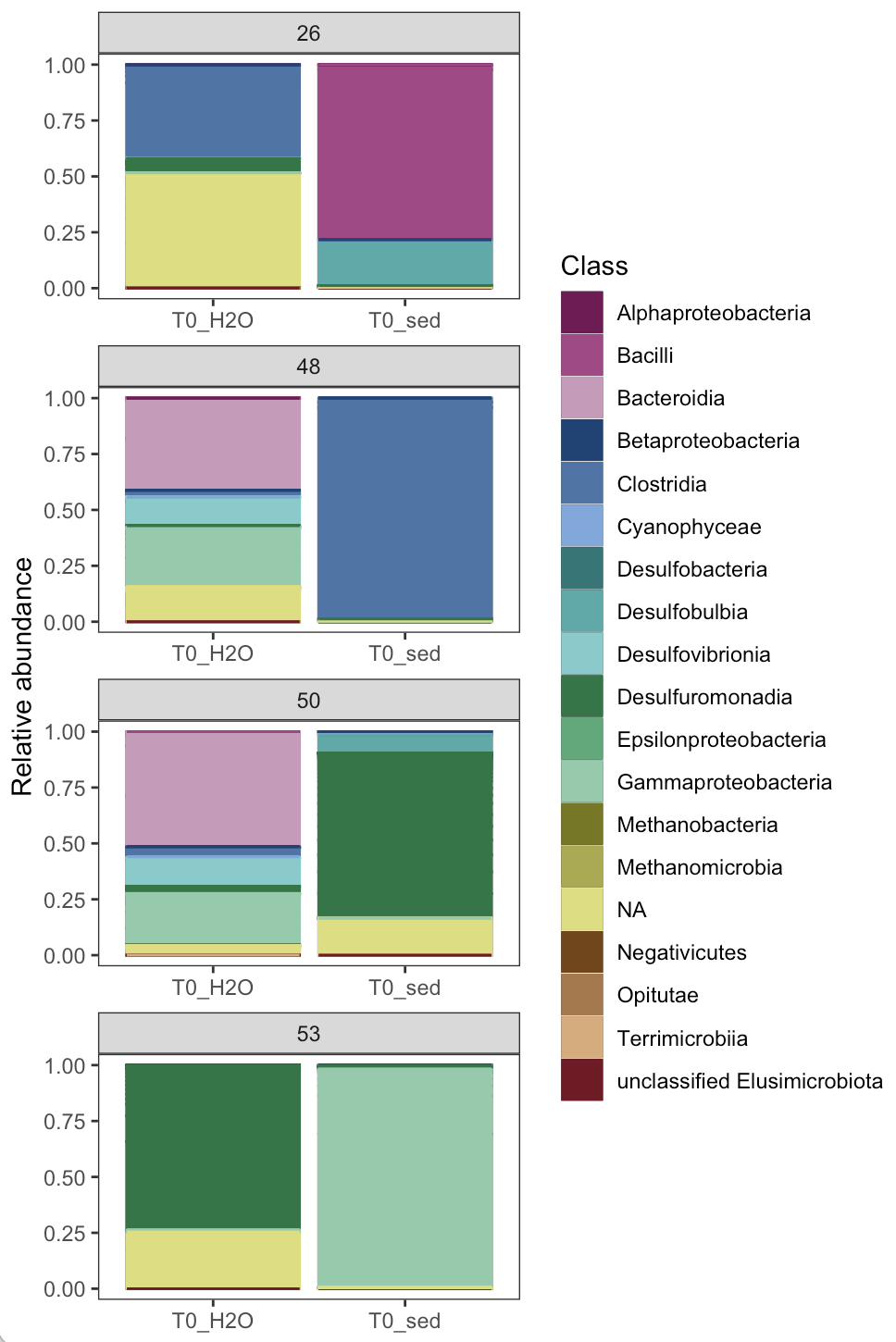


Figure S2. **Diazotroph community composition in water compared to sediment.** The relative abundance of diazotroph classes in sediment (T0_sed) and overlying seawater (T0_H2O) at time 0 (T_0_: time of field sampling and the start of incubation) at each station was assessed through *nifH* amplicon sequencing. NA: not assigned taxonomy at class level. Note that Station 38 is excluded, as no DNA sample from the water was taken there.

Figure S3. **Sediment diazotroph community composition after incubation.** Redundancy analysis of the diazotroph community composition constrained on nitrogen fixation in each incubated vial displaying the top twelve orders. If the group did not have an assigned taxonomy at the order level, the closest above-lying available taxonomic rank is given, e.g. “*Gammaproteobacteria* Class”. The light grey lines from the centre represent the loadings of remaining orders (i.e. beyond the top twelve displayed orders). Dashed lines visualise overlapping labels in the plot and do not represent loadings.

Figure S4. **Partial *nifH* maximum-likelihood phylogenetic tree (amino acid sequences) of the top 20 amplicon sequence variants (ASVs) and reference sequences.** *nifH* clusters/subclusters are denoted as colors (blue: 3C, light green: 3N, orange: 1C, purple: 1A, pink: 1G, dark green: 1G). The accession number and origin of environmental reference sequences are denoted in each node label. Bootstrap values based on 100 iterations are presented as grey quadrats scaled from 50-100%. ASVs from the current study are marked in bold font. Reference sequences originate from [1–6], and the NCBI database (<https://blast.ncbi.nlm.nih.gov/>).

Table S1. **Primers for PCR.** List of primer sequences used to amplify the marker gene *nifH* in the nested PCR [7, 8].

| Primer | Sequence (5'-3') | Reference |
| --- | --- | --- |
| nifH1 | TGYGAYCCNAARGCNGA | Zehr & McReynolds, 1989 |
| nifH2 | ADNGCCATCATYTCNCC |  |
| nifH3 | ATRTTRTTNGCNGCRTA | Zani et al., 2000 |
| nifH4 | TTYTAYGGNAARGGNGG |  |

**References**

1. von Friesen LW, Laber CP, Kristensen BH, Nysted E, Sundbom M, Bertilsson S, et al. From temperate to polar waters: Transition to non-cyanobacterial diazotrophy upon entering the Atlantic gateway of the Arctic Ocean. bioRxiv. 2025.

2. Fernández-Méndez M, Turk-Kubo KA, Buttigieg PL, Rapp JZ, Krumpen T, Zehr JP, et al. Diazotroph Diversity in the Sea Ice, Melt Ponds, and Surface Waters of the Eurasian Basin of the Central Arctic Ocean. Front Microbiol. 2016;7 NOV:1–18.

3. Reeder CF, Arévalo-Martínez DL, Carreres-Calabuig JA, Sanders T, Posth NR, Löscher CR. High Diazotrophic Diversity but Low N_2_ Fixation Activity in the Northern Benguela Upwelling System Confirming the Enigma of Nitrogen Fixation in Oxygen Minimum Zone Waters. Front Mar Sci. 2022;9 May:1–15.

4. Turk-Kubo KA, Gradoville MR, Cheung S, Cornejo-Castillo FM, Harding KJ, Morando M, et al. Non-cyanobacterial diazotrophs: global diversity, distribution, ecophysiology, and activity in marine waters. FEMS Microbiol Rev. 2023;47:1–25.

5. Dang H, Yang J, Li J, Luan X, Zhang Y, Gu G, et al. Environment-dependent distribution of the sediment *nifH*-harboring microbiota in the northern South China Sea. Appl Environ Microbiol. 2013;79:121–32.

6. Shiozaki T, Fujiwara A, Ijichi M, Harada N, Nishino S, Nishi S, et al. Diazotroph community structure and the role of nitrogen fixation in the nitrogen cycle in the Chukchi Sea (western Arctic Ocean). Limnol Oceanogr. 2018;63:2191–205.

7. Zehr JP, McReynolds LA. Use of degenerate oligonucleotides for amplification of the *nifH* gene from the marine cyanobacterium *Trichodesmium thiebautii*. Appl Environ Microbiol. 1989;55:2522–6.

8. Zani S, Mellon MT, Collier JL, Zehr JP. Expression of *nifH* genes in natural microbial assemblages in Lake George, New York, detected by reverse transcriptase PCR. Appl Environ Microbiol. 2000;66:3119–24.
